# Significant improvement of direct reprogramming efficacy of fibroblasts into progenitor endothelial cells by ETV2 and hypoxia

**DOI:** 10.1186/s13287-016-0368-2

**Published:** 2016-08-04

**Authors:** Phuc Van Pham, Ngoc Bich Vu, Hoa Trong Nguyen, Oanh Thuy Huynh, Mai Thi-Hoang Truong

**Affiliations:** Laboratory of Stem Cell Research and Application, University of Science, Viet Nam National University, 227 Nguyen Van Cu, District 5, Ho Chi Minh City, Viet Nam

**Keywords:** Direct reprogramming, Endothelial progenitor cells, Fibroblast, ETV2, Epigenetic reprogramming

## Abstract

**Background:**

Endothelial progenitor cell (EPC) transplantation is a promising therapy for ischemic diseases such as ischemic myocardial infarction and hindlimb ischemia. However, limitation of EPC sources remains a major obstacle. Direct reprogramming has become a powerful tool to produce EPCs from fibroblasts. Some recent efforts successfully directly reprogrammed human fibroblasts into functional EPCs; however, the procedure efficacy was low. This study therefore aimed to improve the efficacy of direct reprogramming of human fibroblasts to functional EPCs.

**Methods:**

Human fibroblasts isolated from foreskin were directly reprogrammed into EPCs by viral ETV2 transduction. Reprogramming efficacy was improved by culturing transduced fibroblasts in hypoxia conditions (5 % oxygen). Phenotype analyses confirmed that single-factor ETV2 transduction successfully reprogrammed dermal fibroblasts into functional EPCs.

**Results:**

Hypoxia treatment during the reprogramming procedure increased the efficacy of reprogramming from 1.21 ± 0.61 % in normoxia conditions to 7.52 ± 2.31 % in hypoxia conditions. Induced EPCs in hypoxia conditions exhibited functional EPC phenotypes similar to those in normoxia conditions, such as expression of CD31 and VEGFR2, and expressed endothelial gene profiles similar to human umbilical vascular endothelial cells. These cells also formed capillary-like networks in vitro.

**Conclusion:**

Our study demonstrates a new simple method to increase the reprogramming efficacy of human fibroblasts to EPCs using ETV2 and hypoxia.

## Background

Endothelial progenitor cells (EPCs) trigger angiogenesis and vasculogenesis, and thus have been used in the treatment of diseases related to ischemia. EPCs have been isolated from peripheral blood [1–5], bone marrow [6–8], and umbilical cord blood [9–12]. Several studies have demonstrated successful preclinical application of EPCs in various disease models, such as rat myocardial infarction [13–15], murine hindlimb ischemia [16], rat ischemic myocardium [17], dermal wound healing [18], and mouse ovarian grafts [19]. A recent study showed that EPC transplantation could increase neovascularization in porcine models [20]. Clinically, EPC transplantation can improve cardiovascular outcomes. In a phase I/II clinical trial of 24 patients [21] followed by a phase IIb trial with 167 patients [22], almost all patients subjected to EPC transplantation showed improved angina frequency and exercise tolerance. In another phase III trial of 444 patients, CD34 cell transplantation could improve the functional capacity in patients with refractory angina [23]. In addition to coronary artery diseases, other pilot studies have also used autologous CD34 to treat critical limb ischemia and showed reduced amputation rates in critical limb ischemic patients [24]. Several studies have also genetically modified EPCs by transfecting cells with genes to increase the treatment efficacy, such as hTERT-transfected EPCs for ischemic myocardium of rats [25], vascular endothelial growth factor (VEGF)-transfected EPCs for myocardial infarction [26], and VEGF and heme oxygenase-1-transfected EPCs for the hindlimb ischemia mice model [27, 28]. Human cord blood endothelial progenitors could also promote postischemic angiogenesis in an immunocompetent mouse model [29].

Successful application of EPC treatments in the clinic, however, has encountered several obstacles, such as limited EPC sources, low numbers of cells, and low proliferation. The number of EPCs in peripheral blood and bone marrow is extremely low [5, 30]. Therefore, deriving endothelial cells as well as EPCs from pluripotent stem cells was studied [31–33]. Although EPCs derived from embryonic stem cells were suggested to be a more promising therapy compared with umbilical cord EPCs [34], this application has not been translated to the clinic owing to the risk of pluripotent stem cells stimulating tumor formation [35, 36]. Current research has thus focused on human fibroblasts (HFs) as a cell source for reprogramming to EPCs.

The first effort towards direct reprogramming of fibroblasts to EPCs was performed by Margariti et al. [37]. The authors developed a method to generate partial-induced pluripotent stem cells by transferring four reprogramming factors (OCT4, SOX2, KLF4, and c-MYC) to HFs for 4 days. These partial-induced pluripotent stem cells were capable of differentiating into endothelial cells in response to defined media and culture conditions [37]. However, although the endothelial cells obtained could not form tumors, these cells carried oncogenes such as *Sox-2* [37]. Li et al. and Han et al. successfully removed the *Sox-2* gene in a revised version of the procedure and instead only used two genes (*Oct4* and *Klf4*) in combination with soluble factors [38] or *Foxo1*, *Er71*, *Klf2*, *Tal1*, and *Lmo2* [39]. These studies used a mixture of factors to induce fibroblasts to EPCs and involved complex procedures with low efficacy.

Recently, ETV2 was reported as a single factor that could induce direct reprogramming of fibroblasts into EPCs [40, 41] and of amniotic cells into EPCs [42]. In fact, ETV2 is a master gene that regulates various signaling pathways and functions as an essential regulator for vasculogenesis and hematopoiesis. ETV2 and GATA2 bind to the promoter of SPI1 and regulate its expression during embryogenesis [43]. ETV2 regulates cardiac development [44], and vascular regeneration [45]. However, the direct reprogramming of ETV2 transduction was low (about 1 %) [41]. Several studies have reported that hypoxia could improve reprogramming of cells. Foja et al. [46] showed that hypoxia improved the reprogramming of MSCs into induced pluripotent stem cells (iPSCs). Adipose stem cells were also stimulated for reprogramming to iPSCs by hypoxia [47]. Hypoxia also enhanced the reprogramming of fibroblasts into iPSCs [48] and dental pulp cells into iPSCs [49]. This study therefore examined the potential enhancement of direct reprogramming efficacy to EPCs by single-factor ETV2 under hypoxia treatment.

## Methods

### Isolation and culture of human dermal fibroblasts and cell culture

Foreskin was collected from a donor who provided a consent form at the hospital. Foreskin was stored in PBS solution at 4 °C and transferred to the laboratory for isolation and culture of fibroblasts, as described in previous studies [50]. Briefly, the samples were cut into small pieces, placed into wells, and allowed to adhere for 5 min at room temperature. DMEM medium supplemented with 10 % FBS, 1× anti-mitotic-mycotic was then added to the wells and the cultures were maintained at 37 °C, 5 % CO_2_. The cultures were subcultured when cells reached 70–80 % confluence. HFs were continuously subcultured to third passages and these cells were used in further experiments. Human umbilical vein endothelial cells (HUVECs) were purchased from Lifetechnologies (code number C0035C; Carlsbad, CA, USA).

### Lentivirus production

The human ETV2 expression vector (pF1KB9707) was purchased from Addgene (Cambridge, MA, USA). ETV2 was cloned into the vector backbone pSIN4-EF1alpha-IRES-Puro (Plasmid #61061; Addgene, Cambridge, MA, USA) to generate pSIN4-EF1a-ETV2-IRES-Puro. All of the coding sequences in the expression vector were confirmed with a GenomeLab System (Beckman Coulter, Brea, CA, USA). The ETV2 vector was then transfected into HEK293T cells along with pCMV-VSV-G-RSV-Rev and pCMV-dR8.2 (Addgene). Eighteen hours after transfection, the medium was replaced with fresh culture medium, and 48 h later the lentivirus-containing medium was collected, passed through a 0.45-μm filter, and concentrated using centrifugation (8400 × *g* at 4 °C for 16 h). The lentivirus pellets were resuspended in PBS at 10^7^ IFUs/ml.

### ETV2 transduction of cells

HFs were plated on 12-well plates at 7 × 10^4^ cells per well and 24 h later were infected with the 10 μl concentrated lentivirus particles with 5 μg/ml protamine. Plates were grouped into two groups: normoxia and hypoxia. The normoxia group was incubated in 20 % O_2_, 5 % CO_2,_ 37 °C, while the hypoxia group was incubated in 5 % O_2_, 5 % CO_2,_ 37 °C. Another 48 h later, cells were washed twice with PBS and then cultured on 6-cm dishes coated with Cellstart (Lifetechnologies) in EGM-2 medium under normoxia or hypoxia conditions.

### VEGF treatment

Anti-VEGF monoclonal antibody was added into the medium at 10 ng/ml before culturing cells under hypoxia conditions. The medium was replaced after 3 days with medium containing an anti-VEGF antibody (P931; Lifetechnologies). In the normoxia group, recombinant protein VEGF at 10 ng/ml was added into the culture medium. The medium was replaced for 3 days with fresh medium containing recombinant VEGF. Cells were then infected with ETV2 virus. After 21 days, the samples were subjected to experimental analyses.

### Quantitative RT-PCR

Total RNA was extracted using Trizol according to the manufacturer’s instructions. PCR analysis was performed using one-step real-time RT-PCR and the SYBR® Green Quantitative RT-qPCR Kit (Sigma-Aldrich, St. Louis, MO, USA) on a Realplex Mastercycler (Eppendorf). Relative gene expression levels were normalized by comparison with GAPDH. Gene-specific primer pairs are presented in Table 1.

**Table 1:** Gene-specific primers used for quantitative RT-PCR

Gene	Forward primer	Reverse primer
*EFNB2*	TCCTCAACTGTGCCAAACCAGACCAA	AGGCCCTCCAAAGACCCATTTGATGT
*EPHB4*	AAGAAAGTTTCGCAGCCGCTGGCTTT	TCATGTGCTGGACACTGGCCAAGATT
*HEY1*	AAATGCTGCATACGGCAGGAGGGAAA	ATAACGCGCAACTTCTGCCAGGCAT
*JAG1*	TTTGGAGCGACCTGTGTGGATGAGA	TGGTGATGCAAGGTCTCCCTGAAACT
*NR2F2*	GGACCACATACGGATCTTCCAAGAGCAA	TTTCCTGCAAGCTTTCCACATGGGCT
*NRP2*	AGGAGCCCTGTGGTTGGATGTATGA	TGTCACTCTGCAGCCGCAAGAAAT
*PROX1*	ACCCGTTATCCCAGCTCCAATATGCT	ATCGTTGATGGCTTGACGTGCGTA
*SOX18*	TGAACGCCTTCATGGTGTGGGCAAA	CGCGTTCAGCTCCTTCCACGCTTT

### Capillary-like structure formation assay

Cells (2 × 10^4^) were seeded on 96-well flat-bottom plates coated with 30 μl Matrigel (Lifetechnologies) and cultured in EGM-2 medium. Eighteen hours after incubation, capillary-like structures were observed under an Axiovert microscope (Carl-Zeiss, Oberkochen, Germany).

### Flow cytometry and cell sorting

Cells were detached using TrypLe (Lifetechnologies), resuspended in PBS-containing 2 % FBS and 2 mM EDTA, and then stained with fluorochrome-leveled mAbs for 20 min on ice. Living cells were identified by 7-AAD exclusion and then analyzed for cell surface marker expression using a FACSCalibur flow cytometer (BD Bioscience). Fifteen and 25 days after lentivirus gene transduction, cells were labeled as already described and sorted using a FACSJazz (BD Bioscience). Collected events were analyzed using FACS software.

### VEGF measurement

The supernatant was collected after changing with fresh medium for 48 h and stored at −20 °C until analysis. VEGF measurement was performed using a human VEGF ELISA kit (Abcam, Cambridge, UK).

### Statistical analysis

Statistical analyses of all endpoints were performed using the two-sided Student’s *t* test or one-way analysis of variance. All data are presented as mean ± SD. *p* < 0.05 was considered statistically significant. Data were analyzed with Prism 6.0 software.

## Results

### Isolation and culture of HFs

**Fig. 1:**
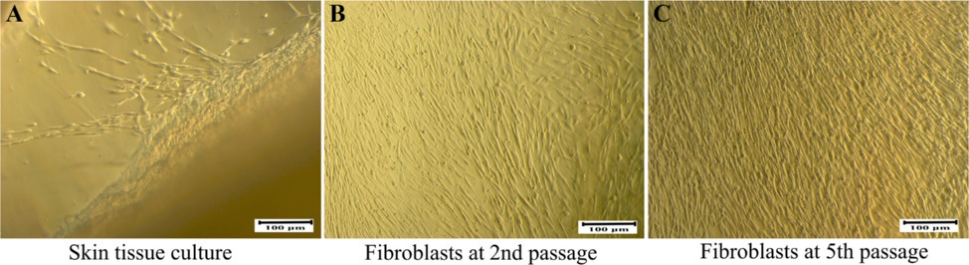
Isolation and culture of HFs from human skin. (**a**) Skin tissue was cultured to isolate the fibroblasts. These fibroblasts were subcultured to the second passage (**b**) and fifth passage (**c**) for further use

Dermal HFs were isolated by culture expansion from dermal tissues. HFs appeared in culture at day 4 with a spindle shape (Fig. 1a). At day 14, the fibroblasts reached 70–80 % confluency (Fig. 1b). The cultures were subcultured to expand the fibroblast population and eliminate keratinocyte contamination. At the fifth passage, the fibroblasts were homogeneous in shape under a microscope (Fig. 1c). These cells were stored in liquid nitrogen for further experiments.

### ETV2 transduced fibroblasts contain CD31-positive cells

**Fig. 2:**
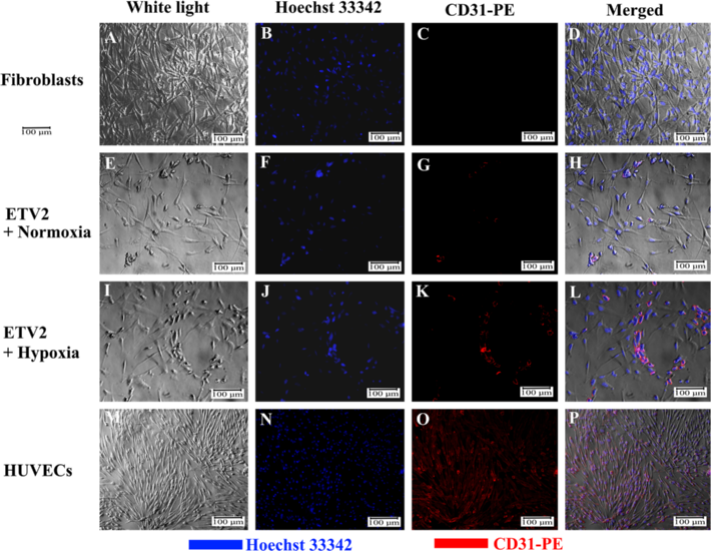
Expression of CD31. HFs did not express the CD31 marker (**a**–**d**). Some cells expressed CD31 in normoxia conditions and transduction with ETV2 (**e**–**h**) and in transduction with ETV2 and hypoxia conditions (**i**–**l**). Almost all HUVECs expressed CD31 (**m**–**p**). *HUVEC* human umbilical vein endothelial cell

To establish EPCs, HFs were transduced with ETV2 virus for 14 days, after which cells were examined for the EPC phenotype. Using an inverted microscope, we observed some clusters of fibroblasts with four to five cells with a round shape (Fig. 2e). We also examined the morphology of cells in both hypoxia and normoxia conditions, and observed more clusters in the hypoxia group (Fig. 2i) compared with the normoxia group.

To confirm these round cells as EPCs, we evaluated CD31 expression. The results showed that round cells in both the normoxia and hypoxia groups were positive for CD31 (Fig. 2e–h and Fig. 2i–l), while spindle-like cells were negative for CD31. Fibroblasts (100 %) were negative for CD31 expression (Fig. 2a–d), while 100 % of HUVECs, used as a positive control, were positive for CD31 expression (Fig. 2m–p).

### ETV2 combined with hypoxia improved reprogramming efficacy compared with ETV2 transduction alone

**Fig. 3:**
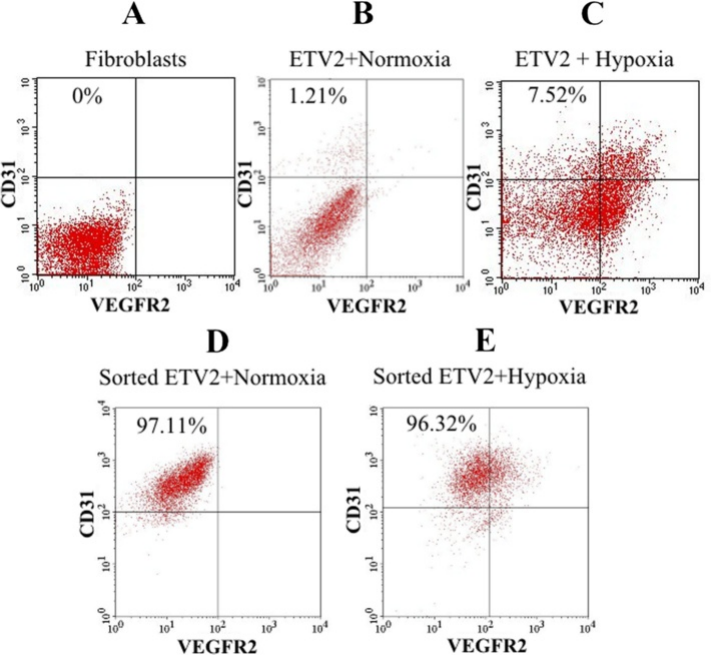
Hypoxia improves the reprogramming of ETV2 of fibroblasts into EPCs. HFs were negative for both CD31 and VEGFR2 (**a**), while ETV2 transduced cells contained a small population of CD31^+^VEGFR2^+/−^ (**b**). In the hypoxia condition, ETV2 transduced fibroblasts significantly increased to form CD31^+^VEGFR2^+^ cells (**c**). To evaluate some cell characteristics from two cell populations, all CD31^+^ cells in the groups of normoxia and transduction with ETV2 (**d**) and of hypoxia and transduction with ETV2 (**e**) were sorted

To compare the effects of normoxia and hypoxia on the efficacy of reprogramming fibroblasts to EPCs, samples transduced for 14 days were analyzed for CD31 expression by flow cytometry. The results showed that ETV2-transduced fibroblasts under both normoxia and hypoxia conditions contained a small population of CD31-positive cells. However, in the hypoxia group the reprogramming efficacy toward EPCs significantly increased to 7.52 ± 2.31 %, compared with 1.21 ± 0.61 % in the normoxia group (*p* > 0.05). We did not observe any CD31-positive cells in the negative control (fibroblasts) (Fig. 3a), and confirmed 100 % of HUVECs positive for CD31 expression. Similarly, ETV2 virally transduced fibroblast samples showed a significant increase in the cell population positive for VEGFR2, and an increase in cells positive for both CD31 and VEGFR2 (Fig. 3b, c).

To evaluate the induced EPCs in both normoxia and hypoxia compared with HUVECs, the CD31-positive populations in both normoxia and hypoxia were sorted using a cell sorter. Induced EPCs were enriched to 97.11 ± 5.17 % and 96.32 ± 4.32 % in normoxia and hypoxia samples at the second sorting times, respectively (Fig. 3d, e).

**Fig. 4:**
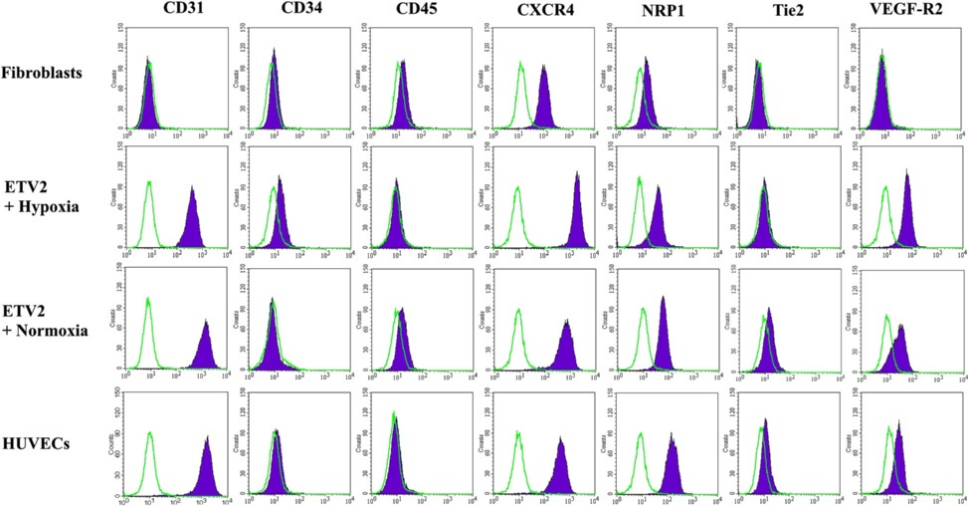
ETV2 transduced fibroblasts expressed EPC phenotypes. The transduced cells from both groups (ETV2 + normoxia and ETV2 + hypoxia) exhibited some similar characteristics. They were positive for CD31, CXCR4, and NRP1. VEGFR2 expression was different in the two groups; in hypoxia conditions the transduced cells strongly expressed this marker compared with normoxia conditions. Generally, ETV2 transduced cells in both normoxia and hypoxia conditions exhibited phenotypes similar to HUVECs and different to fibroblasts. *HUVEC* human umbilical vein endothelial cell

Sorted induced EPCs were stained with markers for EPCs and expressions were assessed by flow cytometry (Fig. 4). HFs (negative control) were negative for CD31, VEGFR2, CD34, Tie2, and CD45, were slightly positive for NRP1 (11.43 %), and were strongly positive for CXCR4 (95.43 %). Sorted induced EPCs under hypoxia conditions were positive for CD31 (100 %), VEGFR2 (95.31 %), NRP1 (88.89 %), and CXCR4 (100 %), and were negative for CD34, Tie2, and CD45. Sorted induced EPCs in normoxia conditions were also positive for CD31 (100 %), VEGFR2 (40.12 %), NRP1 (95.19 %), and CXCR4 (100 %), and were negative for CD34, Tie2, and CD45. The marker profile of induced EPCs in both normoxia and hypoxia conditions were similar to the HUVEC positive control, with positive expression of CD31 (100 %), VEGFR2 (60.67 %), NRP1 (100 %), and CXCR4 (100 %), and negative expression for CD34, Tie2, and CD45. The marker profiles were similar between normoxia-induced EPCs and hypoxia-induced EPCs.

### Similar gene expression patterns in induced fibroblasts and HUVECs

**Fig. 5:**
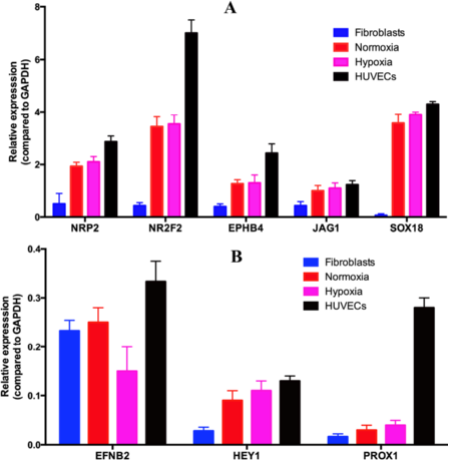
Expression of some genes in induced cells compared with HUVECs. (**a**) *NRP2*, *NR2F2*, *EPHB4*, *JAG1*, and *SOX18.* (**b**) *EFNB2*, *HEY1*, and *PROX2*. Generally almost endothelial cell-related genes significantly increased in ETV2 transduced fibroblasts in both hypoxia and normoxia. However, expression of these genes in transduced cells was lower than in HUVECs. *HUVEC* human umbilical vein endothelial cell

To confirm whether induced EPCs were similar to normal EPCs/endothelial cells (with HUVECs as control), several endothelial cell-related genes were evaluated in induced EPCs in both normoxia and hypoxia conditions compared with HUVECs. The results showed that almost all endothelial cell-related genes, including *NRP2*, *NR2F2*, *EPHB4*, *JAG1*, *EFNB2*, *HEY1*, *PROX2*, and *SOX18*, were expressed in induced EPCs. These genes were expressed at low levels in HFs, and were strongly expressed in HUVECs. Generally, endothelial cell-related genes significantly increased in ETV2 transduced fibroblasts in both hypoxia and normoxia. However, expression of these genes in transduced cells was lower than in HUVECs. There were some differences in expression of some genes between induced EPCs in hypoxia and normoxia conditions, but these differences were not statistically significant (*p* > 0.05), except for the *EFNB2* gene (Fig. 5).

### Formation of capillary-like networks on Matrigel

**Fig. 6:**
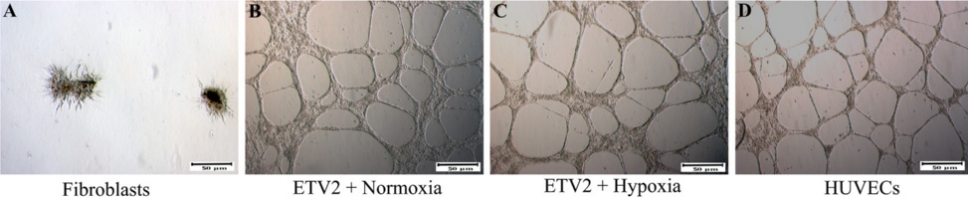
Capillary-like network on Matrigel by cells in groups. (**a**) HFs could not form the capillary-like network. (**b**) ETV2 transduced HFs in both normoxia and hypoxia, successfully forming the capillary-like network (**c**) similar to HUVECs (**d**). *HUVEC* human umbilical vein endothelial cell

Induced EPCs were assessed for in-vitro capillary formation in Matrigel. As shown in Fig. 6, induced EPCs in both normoxia and hypoxia conditions could form capillaries similar to HUVECs, while HFs could not form. There was no difference in capillary formation of induced EPCs in normoxia and hypoxia conditions (Fig. 6).

### VEGF and increased efficacy of reprogramming

We next examined the levels of VEGF in medium from cells cultured in normoxia and hypoxia conditions. The VEGF concentration was significantly increased in the hypoxia culture-derived supernatant compared with the normoxia culture-derived supernatant (5.51 ± 1.43 pg/ml in hypoxia culture vs 1.52 ± 0.41 pg/ml in normoxia culture) (*p* < 0.05).

**Fig. 7:**
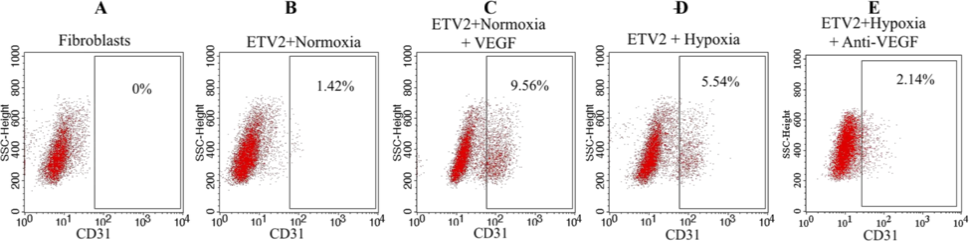
CD31-positive cells analyzed by flow cytometry. As a negative control, HFs did not contain the CD31^+^ population (**a**). In the normoxia condition, ETV2 transduced HFs contained a small CD31^+^ cell population (**b**). A larger population of CD31^+^ cells was present in ETV2 transduced HFs under hypoxia (**d**), similar to the ETV2 transduced HFs under normoxia supplemented with VEGF (**c**). The percentage of CD31^+^ cells significantly decreased in the ETV2 transduced HFs under hypoxia supplemented with anti-VEGF monoclonal antibody (**e**). *VEGF* vascular endothelial growth factor. *SSC* Side Scatter

**Fig. 8:**
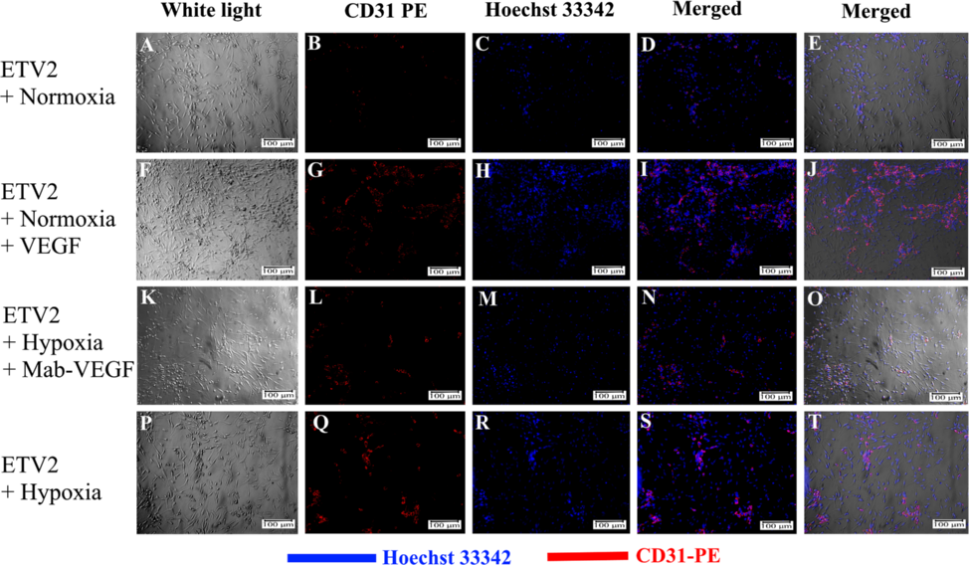
CD31 expression in ETV2 transduced fibroblasts. ETV2 transduction under the hypoxia condition (**p**–**t**) significantly increased CD31^+^ cells compared with normoxia (**a**–**e**), but significantly decreased if the medium was supplemented with anti-VEGF monoclonal antibodies (**k**–**o**). In the normoxia condition, adding VEGF to the medium culture of ETV2 transduced cells could significantly increase the CD31^+^ cells (**f**–**j**). *VEGF* vascular endothelial growth factor

We then examined the effects of modulating VEGF concentrations under both conditions. First, the medium in cells cultured under normoxia conditions was supplemented with VEGF (10 ng/ml) and then fibroblasts were virally transduced with ETV2. Addition of VEGF increased the reprogramming efficacy under normoxia conditions from 1.42 ± 0.32 % of CD31-positive cells in medium without VEGF (Figs. 7b and 8a–e) to 9.56 ± 3.12 % of CD31-positive cells in medium with VEGF (Figs. 7c and 8f–j) compared with 0 % in fibroblasts (Fig. 7a).

We next supplemented cells in hypoxia culture with an anti-VEGF antibody and found that the reprogramming efficacy was lower in anti-VEGF antibody-treated hypoxic cells (2.14 ± 0.49 % CD31-positive cells, Figs. 7e and 8k–o) compared with 5.54 ± 2.13 % CD31-positive cells under hypoxia conditions (Figs. 7d and 8p–t).

## Discussion

EPCs are considered an important cell source for vascular regeneration. However, isolation of EPCs from umbilical cord blood, peripheral blood, and bone marrow has not been successful owing to low numbers of EPCs in these blood sources. Many studies have therefore examined strategies to improve the proliferation and expansion of these cells. Other groups have pursued the direct reprogramming of fibroblasts into EPCs. Recent reports using ETV2 transfection showed that HFs could be directly reprogrammed into endothelial cells or EPCs. However, these preliminary studies showed that reprogramming of fibroblasts to endothelial cells or EPCs was low. In this study, we successfully increased the reprogramming efficacy by hypoxia treatment.

ETV2 overexpression can cause the differentiation of stem cells into endothelial cells. ETV2 is a master gene that regulates various signaling pathways and functions as an essential regulator for vasculogenesis and hematopoiesis. ETV2 interacts with GATA2 in endothelial and hematopoietic cells in the early stages of embryogenesis. ETV2 and GATA2 bind to the promoter of SPI1 and regulate its expression during embryogenesis [43]. Therefore, in a recent study, Liu et al. [51] could induce the hematopoietic and endothelial cell program by overexpression of ETV2. The authors showed that ETV2 could activate ETS genes, resulting in initiation of the hematopoietic and endothelial cell program. ETV2 has also been suggested as a direct regulator of Sox7 [52]. Overexpression of ETV2 in embryonic bodies resulted in enhanced and increased angiogenesis, while knockdown of ETV2 by shRNA significantly decreased angiogenesis [52]. ETV2 also regulates cardiac development [44], and a recent study showed that ETV2 is also an essential factor for vascular regeneration [45].

Elcheva et al. [53] successfully induced human pluripotent stem cells into hematoendothelial cells using ETV2 transfection. Lindgren et al. [54] confirmed that addition of exogenous ETV2 to human ESCs significantly increased the number of cells expressing angioblast genes. Etv2 alone is required for early vasculogenesis, whereas Etv2 and Fli1b function redundantly during late vasculogenesis and early embryonic angiogenesis [55]. With the important role of ETV2 in both early embryogenesis and vascular regeneration in the adult, some initial studies could only use this factor to direct reprogramming of fibroblasts to endothelial cells.

Our results showed that after 14 days of reprogramming with ETV2 transduction, HFs exhibited specific properties of EPCs/endothelial cells, including strong expression of CD31, VEGFR2, and NRP1. Induced EPCs also expressed a gene profile similar to that in HUVECs. More importantly, these sorted induced EPCs with CD31 expression could form capillary-like networks in vitro in Matrigel. Together these phenotypes confirmed that ETV2 transduced fibroblasts were functional EPCs.

Our study also showed that hypoxia markedly affected reprogramming efficacy. Hypoxia treatment during the reprogramming procedure increased the number of cells converted to EPCs. However, hypoxia in combination with ETV2 transfection did not affect the induced EPC phenotypes.

We next considered the mechanisms by which hypoxia could increase the reprogramming of ETV2 transduced fibroblasts into EPCs. We observed a marked difference in VEGF concentration in supernatants from hypoxia and normoxia cultured cells, suggesting that VEGF may be the main factor contributing to the increased efficacy of cell reprogramming. In fact, our results showed that HFs produced over 3-fold more VEGF under hypoxia conditions compared with cells in the normoxia condition, which is similar to previously published studies [56–61]. This observation also was recorded in some stem cells that could increase the production of VEGF under hypoxia [62–64].

To determine whether VEGF is a main factor affecting reprogramming efficacy, we supplemented VEGF in the culture medium under normoxia conditions and found that reprogramming efficacy was increased compared with cells without additional VEGF. Moreover, reprogramming efficacy in hypoxia conditions decreased when cells were supplemented with anti-VEGF monoclonal antibody. We thus concluded that VEGF is the main factor involved in the direct reprogramming of fibroblasts into EPCs by ETV2 transduction.

## Conclusion

EPCs are important cells for angiogenesis and ischemia-related disease treatments. However, EPCs are rare cells in peripheral blood, bone marrow, and umbilical cord blood. Here, we showed that single-factor ETV2 transduction was a simple approach to produce EPCs from fibroblasts. Notably, ETV2 transduction in combination with hypoxia treatment resulted in increased efficacy of reprogramming from fibroblasts to EPCs. These induced EPCs exhibited EPC phenotypes and could form capillary networks in vitro, similar to human umbilical endothelial cells. These findings suggest a simple strategy to increase the reprogramming efficacy from fibroblasts to EPCs.

## Abbreviations

CD, cluster of differentiation; EPC, endothelial progenitor cell; ETV2, E26 transformation-specific Ets Variant 2; HF, human fibroblast; HUVEC, human umbilical vein endothelial cell; iPSC, induced pluripotent stem cell; PBS, phosphate-buffered saline; VEGF, vascular endothelial growth factor; VEGFR2, vascular endothelial growth factor receptor 2
